# General Health Among Parents Who Lost Their Children in the Bam Earthquake

**DOI:** 10.5539/gjhs.v7n5p251

**Published:** 2015-03-16

**Authors:** Masoumeh Rashidinejad, Mahlagha Dehghan, Batool Tirgari, Hossein Rafiei, Sedigheh Iranmanesh

**Affiliations:** 1 Medical Surgical Department, Nursing and Midwifery School, Kerman Medical Science University, Kerman, Iran; 2 Medical Surgical Department, Nursing and Midwifery School, Qazvin University of Medical Science, Qazvin, Iran

**Keywords:** Bam earthquake, children loss, parents, general health

## Abstract

**Aim:**

Bam earthquake and its profound tragedy of thousands killed has caused emotional and psychological trauma for tens of thousands of people who have survived. This study aimed to investigate general health of parents who lose their children in Bam earthquake 10 years after the earthquake.

**Method:**

General health of 166 parents who lost their children in Bam earthquake was assessed using a translated version of General Health Questionnarie-28 items.

**Results:**

The mean score of GHQ was 25.63 ± 15.28. Among all domains, the higher mean score belonged to the category of “anxiety/insomnia” and the lower one belonged to the category of “severe depression. The results revealed significant correlation between total GHQ mean score and gender as well as educational level (0.003)

**Conclusion:**

Providing reflective narrative environments in which survivors can express their own experiences and feelings about earthquake, their loss and how they cope with it seems to be as an effective approach to identify their psychosocial situation and its influential factors. In such narrative environments special attention should be given to older participants, females and those who are single.

## 1. Introduction

Each year earthquake affects large numbers of people throughout the world. This traumatic experiences may result in a wide range of mental and physical health consequences. The earthquake often result in negative income shocks due to loss of closed family members, and have an effect on intra household allocation decisions (Hermida, 2010). Since bereavement is stressful whenever it occurs, the greatest stress, and often the most enduring one, occurs for parents who experience the death of a child. Death of a child is the most significant and traumatic death of any family member (Wang, 2012). According to Xu et al. (2014), direct exposure to natural disasters and the bereavement of a child are each extremely stressful life events and have long-term health consequences, especially increased vulnerability to mental health problems including depression and anxiety (Xu, Herrman, Bentley, Tsutsumi, & Fisher, 2014). According to Seecharan et al. (2004) mothers who experienced the sudden death of their child had somewhat more intense grief reactions than those whose child died of a chronic condition (Seecharan, Andresen, Norris, & Toce, 2004).

Review of literature showed several studies that assessed the effect of natural disaster on psychological or general health of survivors in different countries. In 2012, Chan et al. examined prevalence rates of clinically significant PTSD and depressive symptoms among bereaved adults and non-bereaved adult survivors of 2008 Sichuan earthquake (China). The estimated rates of PTSD and depressive symptoms were 65.6% and 64.8% for those who lost first-degree family members, 34.1% and 45.5% for those who lost second-degree relatives, and 27.1% and 37.5% for non-bereaved survivors respectively. Loss of a child was a significant predictor of psychopathological symptoms (Chan et al., 2012). Xu (2012) also assessed mental health morbidity among women thirty months after the death of a child in the 2008 Sichuan, China earthquake. They found that 85.4% of the women were experiencing symptoms of more than one psychological disorder. In 2011, Priebe et al., assessed the prevalence of mental disorders, level of psychological symptoms and subjective quality of life among a random sample in a rural region in Italy 8 years after an earthquake. They reported that there is no evidence that the earthquake had a negative impact on the mental health of the affected population years later. Johannesson et al. (2011) examined prolonged grief among traumatically Swedish bereaved relatives exposed and none exposed to the 2004 Indian Ocean tsunami in Southeast Asia. They found that tsunami cause prolonged grief, posttraumatic stress reactions, and doubled the risk for impaired mental health (Johannesson, Lundin, Hultman, Fröjd, & Michel, 2011). Within a review of evidence Xu et al. (2013) examined psychological and social consequences of losing a child in a natural or human-made disaster. They concluded that bereaved parents had more mental health problems than bereaved spouses and non-bereaved parents.

There are several studies that assessed the correlation between demographic factors of earthquakes’’ survivors and their general health status. Zhou et al. (2013) found that low school education and witness someone die are also important risk factors for PTSD and depression. The other found relationship between gender and prolonged grief (Johannesson et al., 2011), PTSD (Nygaard & Heir, 2012; Zhou et al., 2013), depression (Zhou et al., 2013), vulnerable to mental health problems (Xu et al., 2013), as well as psychological distress (Montazeri et al., 2005, Nakamura et al., 2014). Age is another factor that correlated with quality of life (QOL), the psychological wellbeing (Cofini, Carbonelli, Cecilia, & di Orio, 2014), PTSD, depression (Wang, 2012; Zhou et al., 2013), as well as psychological distress (Nakamura et al., 2014) among survivors of earthquakes.

Iran [the Islamic Republic] located in Central Eurasia, with a population over 70 million. It is exposed to a wide range of natural and man-made hazards. Totally 181 disasters are recorded in Iran for 1900-2007 period, which caused 155,811 deaths, 168,217 injured and 44,037,516 affected (Ardalan et al., 2009; Ardalan et al., 2011). An earthquake measuring 6.3 on the Richter scale struck the city of Bam in Iran on the 26th of December 2003 at 5.26 A.M. Bam is situated in a desert environment on the southern edge of the Iranian high plateau. The earthquake was devastating, the city was destroyed, left over 40,000 dead and around 30,000 injured, as well as destroying approximately 20,000 homes, leaving more than 45,000 people homeless (Montazeri et al., 2005). The profound tragedy of thousands killed has caused emotional and psychological trauma for tens of thousands of people who have survived (Montazeri et al., 2005). In general earthquakes are known to be related to increased psychological symptoms among survivors and in particular it has been shown that earthquakes might cause PTSD (Montazeri et al., 2005). There are several studies that examined psychological situation of people living in Bam after its huge earthquake. Using a multi-stage stratified sampling Montazeri et al. (2005) assessed psychological distress among the survivors of Bam earthquake in Iran. The findings showed that 58% of the respondents suffered from severe mental health and this was three times higher than reported psychological distress among the general population. There were significant differences between sub-groups of the study sample with regard to their psychological distress. Ardalan et al. (2011), assessed post-disaster quality of life among older survivors five years after the Bam earthquake. Earthquake had negative effect on psychological dimensions and positive effect on social relationships. In the review of literature no study was found to assess general health among parents who lost their children in the Bam earthquake. This study thus was conducted to fulfill this aim 10 years after the earthquake.

## 2. Methods and Materials

### 2.1 Design

The study employed a descriptive-cross sectional design and was conducted in Bam city in South-East of Iran 10 years after the earthquake.

### 2.2 Sample

A convenience sample of 166 parents who lost their children in Bam earthquake participated in this study. The size of sample was selected based on previous published research (Ardalan et al., 2011; Cofini, Carbonelli, Cecilia, & di Orio, 2014).

### 2.3 The instruments

#### 2.3.1 Socio-Demographic Characteristics

First, a questionnaire was designed to obtain background information which was assumed to influence general health. It included questions about gender, age, marital status, educational level, number of lost and survivor children.

#### 2.3.2 The General Health Questionnaire

Assessment of mental health states was done using the Iranian version of the General Health Questionnaire-28 (GHQ-28) (Noorbala, Bagheri Yazdi, & Mohammad, 2009). The GHQ developed as a screening tool to recognize those likely to have or be at risk of developing psychiatric disorders. It was designed to measure common mental health problems and has 4 subscales including: 1) severe depression, 2) anxiety, 3) somatic symptoms, and 4) social withdrawal. Each subscale consists of seven questions. Of the various versions (60, 30, 28 and 12 items), the 28-item version is the one generally used. Each item is assessed using a four-point Likert scale (0 to 3). The total possible score on the GHQ-28 ranges from 0 to 84. The total possible score of each subscale ranges from 0 to 21. The 28-item version classifies any score exceeding the threshold value of 23 as achieving “psychiatric caseness” (Noorbala et al., 2009).

#### 2.3.3 Validity and Reliability of Instruments

Noorbala et al. (2009) validated the Persian version of GHQ-28 in Iran. They found that the GHQ-28 has acceptable validity and reliability. In the following study the internal consistency of GHQ was 0.92 and for four subscales were 0.81 (somatic symptoms), 0.84 (anxiety/insomnia), 0.73 (social dysfunction) and 0.88 (severe depression) respectively.

### 2.4 Data Collection and Analysis

Accompanied by a letter including some information about the aim of the study, the questionnaires were handed out by the first author to 182 parents who lost their children in Bam earthquake [Dec 2013/Feb 2014] at city of Bam. Some oral information about the study was also given as well by the first author. Participation in the study was voluntary and anonymous. 166 of 182 questionnaires were returned with a drop out of 16. Less than 5% of subjects were parents of the same children. In all collected data, 80 % of all questions were answered. Data from the questionnaires were analyzed using the Statistical Package for Social Scientists [SPSS ver. 19]. Descriptive statistics of the sample and measures that were computed included frequencies, means and reliability. ANOVA and Eta squared were used to examine the relationship between the measured factors [demographic variables] and the GHQ scores.

## 3. Results

### 3.1 Socio-Demographic Data

Based on the results, the mean age of participants was 53.94 ± 11.92. Less than 50% were male. Most of parents lost one or two children (73%). Less than 20% no children remained. Nearly 21% of parents were remarriage. Approximately less than 30% were illiterate (Table 1).

**Table 1:** Demographic characteristics of study participants (n=166)

Variable	Frequency (%∗)
Gender Male Female	69 (42.6) 93 (57.4)
Age (yr) ≤ 40 yr > 40 yr	26 (15.7) 140 (84.3)
Children loss (no) One child Two children Above two children	58 (35.6) 61 (37.4) 44 (27.0)
Children residual (no) Yes No	135 (83.9) 26 (16.1)
Remarriage Yes No	35 (21.6) 127 (17.4)
Education statues Illiterate Under diploma & Diploma Above diploma	42 (26.1) 89 (55.3) 30 (18.6)

* Valid percent	

### 3.2 Descriptive Findings

According to the results, the GHQ score ranged from 5 to 84. The mean score of GHQ was 25.63 ± 15.28. Using value of 23 as cut of point, the presence of distress or caseness was 44.6% (n = 74). The means and standard deviations of the GHQ subscales were as follows: 1) somatic symptoms: 6.99 ± 5.13, 2): anxiety/insomnia; 7.70 ± 5.67, 3) social dysfunction: 6.04 ± 3.29, and 4) severe depression: 4.92 ± 4.58. Among all domains, the higher mean score belonged to the category of “anxiety/insomnia” and the lower one belonged to the category of “severe depression” (Table 2).

**Table 2:** The general health mean scores and Standard deviation among study participants

GHQ subscales	Mean	Standard deviation
Somatic symptoms	6.99	5.13
Anxiety/insomnia	7.70	5.67
Social dysfunction	6.04	3.29
Severe depression	4.92	4.58
Total	25.63	15.28

### 3.3. Correlations

The results revealed significant correlation between total GHQ mean score and gender as well as educational level (0.003). A correlation was found between “somatic symptoms” subscale and gender (P = 0.04) as well as educational level (P = 0.001). The category of “anxiety/insomnia” had significant correlation with gender and education (P = 0.004, P = 0.005 respectively), social dysfunction subscale had significant correlation with remarriage (P = 0.04). The severe depression subscale showed a significant correlation with gender (P=0.02) (Table 3).

**Table 3:** Correlation between demographic variables and general health domains

Variables	Somatic symptoms		Anxiety/insomnia		Social dysfunction		Severe depression		Total	

	Mean/SD	P value	Mean/SD	P value	Mean/SD	P value	Mean/SD	P value	Mean/SD	P value
Gender									
Male	5.88 (4.42)	f = 4.35	6.13 (4.75)	f = 8.33	5.35 (2.66)	f = 4.97	3.97 (3.88)	f = 5.66	21.28 (12.04)	f = 8.99
Female	7.54 (5.43)	η^2^= 0.03	8.63 (5.93)	η^2^= 0.05	6.48 (3.59)	η^2^= 0.03	5.70 (5.0)	η^2^= 0.03	28.37 (16.67)	η^2^= 0.05
		P = 0.04		P = 0.004		P = 0.03		P = 0.02		P = 0.003

Age									
≤ 40	6.25 (3.55)	f = 0.4	6.75 (3.78)	f = 0.64	5.38 (3.29)	f = 1.05	5.12 (4.03)	f = 0.03	23.50 (11.53)	f = 0.43
> 40	6.96 (5.31)	η^2^= 0.003	7.74 (5.85)	η^2^= 0.004	6.11 (3.27)	η^2^= 0.007	4.94 (4.74)	η^2^= 0.00	25.73 (15.85)	η^2^= 0.003
		P = 0.53		P = 0.43		P = 0.31		P = 0.86		P = 0.51

Children loss									
One child	5.74 (4.05)	f = 2.35	6.19 (4.12)	f = 2.85	5.28 (2.46)	f = 2.44	4.09 (3.74)	f = 1.81	21.29 (9.91)	f = 3.46
Two children	7.62 (5.81)	η^2^= 0.03	8.48 (6.27)	η^2^= 0.03	6.51 (3.78)	η^2^= 0.03	5.68 (5.18)	η^2^= 0.02	28.20 (18.06)	η^2^= 0.04
Above two children	6.89 (5.10)	P = 0.1	7.56 (5.57)	P = 0.06	6.02 (3.27)	P = 0.09	4.94 (4.62)	P = 0.17	25.39 (15.21)	P = 0.03

Children residual									
Yes	5.81 (3.24)	f = 1.54	6.88 (4.62)	f = 0.54	6.19 (4.06)	f = 0.06	5.50 (5.63)	f = 0.41	24.38 (14.28)	f = 0.18
No	7.16 (5.37)	η^2^= 0.01	7.76 (5.74)	η^2^= 0.003		η^2^= 0.000		η^2^= 0.003	25.78 (15.42)	η^2^= 0.001
		P = 0.23		P = 0.46	6.02 (3.12)	P = 0.81	4.87 (4.43)	P = 0.52		P = 0.67

Remarriage									
Yes	5.71 (3.75)	f = 2.14	6.71 (4.09)	f = 0.86	5.00 (2.53)	f = 4.23	4.46 (3.63)	f = 0.34	21.86 (8.79)	f = 2.09
No	7.10 (5.26)	η^2^= 0.013	7.69 (5.80)	η^2^= 0.005		η^2^= 0.026		η^2^= 0.002	25.89 (15.67	η^2^= 0.013
		P = 0.15		P = 0.36	6.19 (3.15)	P = 0.04	4.95 (4.66)	P = 0.56		

Education statues									
Illiterate	8.55 (6.22)	f = 7.87	8.21 (6.43)	f = 5.52	6.81 (3.95)	f = 2.95	5.90 (5.02)	f = 1.56	29.48 (18.08)	f = 5.88
Under diploma & diploma	6.98 (4.62)	η^2^= 0.09	8.17 (5.31)	η^2^= 0.07	5.83 (2.56)	η^2^= 0.04	4.53 (4.18)	η^2^= 0.02	25.51 (13.08)	η^2^= 0.07
Above diploma	4.03 (2.34)	P = 0.001	4.60 (3.23)	P = 0.005	5.10 (2.78)	P = 0.06	4.41 (4.35)	P = 0.21	18.00 (9.92)	P = 0.003

## 4. Discussion

Earthquakes can damage and reduce the health of survivors. This research revealed that the presence of mental health problem among parents who lost children in Bam earthquake is about 45%. In 2005 Montazari et al. examined general health of survivors 2 years after Bam earthquake. They reported that 58% of the respondents suffered from severe mental health and this was three times higher than reported psychological distress among the general population. The difference between the result of following study and Montazari et al. (2005) study could be related to the time of conducting studies. The following study conducted 10 years after Bam earthquake whereas their study was done 2 years after earthquake. Time could be one of the factors that positively affect survivors’ coping mechanism. The distress and anxiety of survivors may decrease by the time (Videbeck, 2013). According to Nakamura et al., psychological distress after the 2004 Niigata-Chuetsu Earthquake in Japan, were 51% that decreased to 33.8% after 5 years follow up (Nakamura, Kitamura, & Someya, 2014). The other factor that may cause the difference between the results of two studies could be related to the type of sample using in two studies. The sample in Montazeri’s et al., (2005) study consists of elderly survivors of Bam earthquake whereas the sample of following study consists of parents who lost their children in the same earthquake. Culturally, it seems that people in Bam are reluctant to reveal their psychological problems and are not likely to refer to the psychiatrist to receive mental care in order to reduce the negative impact of disaster on their mental health. Xu et al. (2012), also indicated a high prevalence rate of depression and PTSD (82.3%, 82.3%) among women survived from Sichuan, China earthquake.

The results of this study revealed that among all categories of the scale, the lowest mean score belonged to the category of “severe depression”. According to Person et al. (2006), the prevalence of depression within an entire community affected by mass disaster is still largely unknown. Salcioğlu et al. (2003) stated that research about earthquake survivors after the Marmara earthquake in Turkey suggested that the rates of major depression in earthquake survivors ranged from 13% to 22% (Rowlands, 2012). The figure for major depression among survivors after the Armenian earthquake was 18% (Armenian et al., 2002). Whereas severe depression obtained the lowest mean score among all categories of GHQ scale, the highest mean score belonged to the category of the subscale of anxiety. Anxiety is a tense unsettling anticipation of a threatening but formless or vague event, a feeling of uneasy suspend, whereas depression is a mood disorder that causes a persistent feeling of sadness and loss of interest (B. J. Sadock & V. A. Sadock, 2010). Bam earthquake was one of the greatest disasters in the world therefore the survivors were faced with major PTSD. PTSD is an anxiety disorder that can develop after an individual has experienced or witnessed a major trauma (B. J. Sadock & V. A. Sadock, 2010).

This study revealed a significant correlation between total GHQ mean score as well as category of somatic symptom and gender. It means that women had worse general health compared to men. Earlier studies that conducted in different context revealed that females had prolonged grief (Johannesson et al., 2011), PTSD (Nygaard & Heir, 2012), more depression (Zhou et al., 2013), are more vulnerable to mental health problems (Xu et al., 2013), and have more psychological distress (Montazeri et al., 2005; Nakamura et al., 2014; Xu et al., 2014) compared to males. Female survivors seems to have negative attitude towards traumatic events than males, are more likely to express certain anxiety symptoms than males, and may view themselves as incompetent individuals compared to the males (Tolin & Foa, 2002). Moreover, in Middle Eastern culture males are appreciated for their patience, resistance, and struggles, but females should be supported to release their distress and sorrows, they are permitted to show their weakness or to lack control and they can demand their needs (Salimi, Gomashchi, & Taghavi, 2008).

The results of following study indicated a positive correlation between educational level and general health status. This finding could be supported by the results of earlier studies where they found that lower education was related to higher PTSD, depression (Goenjian et al., 2011), low quality of life (Valenti et al., 2013), and psychosocial distress (Montazeri et al., 2005) among earthquakes’ survivors. Beverly et al. (2013) conducted a review of 25 years of evidence about the relationship of adolescents’ academic achievement and health behaviors in the United States. They found that educational attainment is interrelated with health behaviors.

This study showed significant correlation between marital status and social dysfunction. It means that women who had remarriage have better social function than those who had not. Bradbury and Karney (1992) stated that remarriage was associated with greater socioeconomic security and life satisfaction compared to remaining divorced or separated. In general, married people are healthier than those who are not married across a wide array of health outcomes (Schoenborn, 2004). Marriage also may provide an emotionally fulfilling, intimate relationship, satisfying the need for social connection, which could have implications for both physical and mental health (Wood, Goesling, & Avellar, 2007).

A positive correlation was found between children loss and general health. It means that parents who lost more than one child had worse general health status compared the other groups. Xu et al. (2013) reported that parents who have lost children in disasters are at high risk of mental health problems. Having other children at the time of death and giving birth to a new child after the death may induce a sense of purpose and give life meaning in parents’ life. The prevalence of psychological symptoms was higher in mothers who did not have a child after losing the first one. Feelings of loneliness or meaningless without the person who died makes mothers confuse about their roles in life or diminished their sense of self (i.e. feeling that a part of one self has died) (Arnold, Gemma, & Cushman, 2005).

This study has several limitations, the most critical being the absence of a control group of non-earthquake-affected parents with child loss, which made it difficult to associate GHQ outcomes with the earthquake and resulting traumas. The lack of baseline GHQ data in the earthquake-affected area to compare the post-earthquake GHQ with the following situation is another limitation. The study was limited by its cross-sectional design. The lack of longitudinal tracking meant that no data were captured on parents who died, immigrated to other parts of Iran, or were admitted to nursing homes. There are also important predictive demographic variables such as presence of psychiatric illness/or family psychiatric illness that were not included and examined in this study. Further researches are needed to assess the effect of such variables on general health of Bam earthquake’s survivors.

## 5. Conclusions

According to the finding of this study, even after 10 years of Basm earthquake, there are still challenges regarding survivors’ psychological situation. Participants may have no contact with psycho-social counselors because they are not familiar with such counseling services or these services are perceived as stigma. Creating friendly meetings to assess informally their situation and make them aware of psycho-social assistance services may help them gain access to all required services. Some educational programs aimed at raising survivors’ self-awareness of their psychological situation, accompanied by interventions intended to decrease their psychological impairments seems necessary to improve the quality of life of survivors. Providing reflective narrative environments in which survivors can express their own experiences and feelings about earthquake, their loss and how they cope with it seems to be as an effective approach to identify their psychosocial situation and its influential factors. Exposure to such narratives under psychiatric supervision may provide survivors appropriate opportunities to learn from each other and see meaning, personal growth despite their losses and pain. In such narrative environments special attention should be given to older participants, females and those who are single. Long-term plans and psychosocial promotion activities are needed to continuously educate survivors about mental health and psychosocial well-being in public places such as schools, local organizations and religious services. Since QOL and its domains especially its psychological domain is multidimensional and cultural base, it is suggested to conduct some appropriate qualitative studies to explore survivors’ experiences in the context and then develop a valuable instrument regarding their QOL in order to appropriately assess it.
